# Risk Factors Associated With Clostridium difficile-Associated Diarrhea

**DOI:** 10.7759/cureus.18115

**Published:** 2021-09-20

**Authors:** Aarzoo Gupta, FNU Savanti, Balvender Singh, Priyanka Sachdev, Deepak Raj, Ishan Garg, Suraj K Aruwani, Faizan Shaukat

**Affiliations:** 1 Internal Medicine, Safdarjung Hospital, Faridabad, IND; 2 Internal Medicine, Liaquat National Hospital and Medical College, Karachi, PAK; 3 Internal Medicine, Ghulam Mohammad Mahar Medical College, Sukkur, PAK; 4 Internal Medicine, Liaquat University of Medical and Health Sciences, Jamshoro, PAK; 5 Internal Medicine, Ross University School of Medicine, Miami, USA; 6 Internal Medicine, Jinnah Sindh Medical University, Karachi, PAK; 7 Internal Medicine, Dow University of Health Sciences, Karachi, PAK

**Keywords:** clostridium difficile, diarrhea, gastrointestinal symptoms, cdi, spore forming bacilli

## Abstract

Introduction: Recent years have been alarming due to the sudden, dramatic rise in the incidence of Clostridium difficile infection (CDI). Identifying and addressing the risk factors associated with CDI will help in reducing the incidence of infection and associated complications.

Methods: This case-control study was conducted in a tertiary care hospital in Pakistan from June 2020 to March 2021, in which 200 patients diagnosed with Clostridium difficile-associated diarrhea (CDAD) were enrolled in the study. CDAD was diagnosed based on clinical symptoms and stool enzyme immunoassay. Another 200 participants without a diagnosis of CDAD were enrolled from the outpatient department as a control group. Participants were enrolled after seeking informed consent.

Results: In patients older than 65, risk of CDI was higher compared to participants lower than 65 years old (15.5% vs. 8.0%; p value: 0.02). Hospitalization (25.5% vs. 6.0%; p value < 0.0001), the use of proton pump inhibitors in last 30 days (23.0% vs. 10.5%; p value: 0.001) , and use of antibiotics in the last 30 days (36.0% vs. 10.5%; p value < 0.0001) were significantly higher in participants with CDI.

Conclusion: Hospitalization, the usage of proton pump inhibitors, and antibiotics in the last 30 days were significantly associated with CDI. A higher incidence of CDI was associated with risk factors like increased body mass index, diabetes, chronic kidney disease, and malignancy.

## Introduction

Clostridium difficile (C. difficile) is an omnipresent gram-positive, spore-forming bacillus that causes gastrointestinal symptoms, ranging from benign afebrile or febrile diarrhea to violent colitis, severe sepsis, toxic megacolon, and organ perforation to even death [1]. A recent meta-analysis, which included 51 studies, demonstrated that in Asia the proportion of CDAD in patients with nosocomial diarrhea was 14.8% and the related mortality was found to be 8.9% [2].

Suárez-Bode et al. in their 2019 study indicated that in recent years there has been an increase in the incidence of C. difficile infection (CDI) both in the hospital and community settings [3]. The growing prevalence of CDI in hospitalized patients has led to a global burden with rising mortality, morbidity, health care costs, and hospital stay despite adequate awareness of risks associated with excessive use of broad-spectrum antibiotics and the importance of improving hospital and environmental hygiene [4]. Broad-spectrum antibiotics and proton pump inhibitors (PPIs) are the main contributors for causing the recurrence of Clostridium difficile-associated diarrhea (CDAD) by disrupting the normal gut flora and reducing the stomach acid secretion that is the main host defense mechanism against ingested C. difficile and its spores [4].

Aging; comorbidities; use of antibiotics, PPIs, and histamine-2 receptor antagonists (H2RA); exposure to health care settings; and obesity are among the commonly reported risk factors [5-7]. Other risk factors include the use of non-steroidal anti-inflammatory drugs, altered vitamin D levels, and genetics [8]. Globally studies have been done to determine risk factors associated with CDI; however, very little work is done in developing countries. Identifying and addressing these risk factors will help in reducing the incidence of infection and complications associated with it.

## Materials and methods

This case-control study was conducted in a tertiary care hospital in Pakistan. It was conducted from June 2020 to March 2021, in which 200 patients diagnosed with CDAD were enrolled in the study (n=200) via consecutive convenient non-probability sampling. Patients were enrolled after obtaining informed consent. Ethical review approval was taken from Liaquat University of Medical and Health Sciences before enrollment of patients (LUMHS/2020/ERC-16). CDAD was diagnosed based on clinical symptoms (diarrhea) and stool enzyme immunoassay [9]. Another 200 participants without a diagnosis of CDAD were enrolled from the outpatient department as a control group.

Patients’ detailed history, including the use of antibiotics, PPI, and H2RA, previous history of CDI, and hospitalization in the previous 30 days were noted in a self-structured questionnaire. Patients’ body mass index (BMI) was calculated and noted in a questionnaire. Further information related to comorbidities, like diabetes, chronic kidney disease (CKD), hypertension, and malignancy was also noted in a self-structured questionnaire.

Statistical analysis was done using the Statistical Package for Social Sciences® software, version 23.0 (SPSS; IBM Corp., Armonk, NY, USA). Categorical data such as age distribution was presented as frequency and percentage. Chi-square was applied to compare the risk factors for both groups. The odds ratio was calculated using an online calculator (medCalc; www.mdcalc.com). A p-value of less than 0.05 meant that the difference between the groups is significant and the null hypothesis is void.

## Results

In patients older than 65, risk of CDI was higher compare to participants lower than 65 years old (15.5% vs. 8.0%; p value: 0.02). Hospitalization (25.5% vs. 6.0%; p value < 0.0001), the use of proton pump inhibitors in last 30 days (23.0% vs. 10.5%; p value: 0.001) , and use of antibiotics in the last 30 days (36.0% vs. 10.5%; p value < 0.0001) were significantly higher in participants with CDI. A higher incidence of CDI was associated with risk factors like increased BMI, diabetes, chronic kidney disease (CKD), and malignancy (Table 1). 

**Table 1:** Comparison of risk factors between both groups BMI: body mass index, CKD: chronic kidney disease, H2RA: histamine–2 receptor antagonists, PPI: proton pump inhibitor

Risk factors	Case group (n=200)	Control group (n=200)	Odds ratio	p-value
Age greater than 65	31 (15.5%)	16 (8.0%)	2.10 (1.11-3.99)	0.02
Male	98 (49.0%)	100 (50.0%)	1.04 (0.70-1.54)	0.84
Hospitalization in last 30 days	51 (25.5%)	12 (6.0%)	5.36 (2.75-10.42)	<0.0001
Antibiotics in last 30 days	72 (36.0%)	21 (10.5%)	4.76 (2.80-8.19)	<0.0001
PPIs in last 30 days	46 (23.0%)	21 (10.5%)	2.54 (1.45-4.45)	0.001
H2RA in last 30 days	32 (16.0%)	20 (10.0%)	1.71 (-.94-3.11)	0.07
BMI more than 25 kg/m2	62 (31.0%)	42 (21.0%)	1.69 (1.07-2.65)	0.02
Diabetes	54 (27.0%)	32 (16.0%)	1.94 (1.18-3.17)	0.008
Hypertension	31 (15.5%)	29 (14.5%)	1.08 (0.62-1.87)	0.77
CKD	38 (19.0%)	19 (9.5%)	2.23 (1.23-4.03)	0.007
Malignancy	12 (6.0%)	4 (2.0%)	3.12 (0.99 to 9.86)	0.05
Hypercholesterolemia	22 (11.0%)	20 (10.0%)	1.08 (0.57-2.06)	0.79

## Discussion

In our study, participants of the case group with ages greater than 65 were significantly more compared to the control group. Literature has shown that patients aged more than 65 are five to 10 times at an increased risk of CDI. This age group is not only termed as a risk factor for CDI, such patients also demonstrate poor prognosis, leading to increased clinical severity and death rates [10,11]. CDI was also observed to be positively related to the recent hospitalization. A majority of the CDI cases are linked with being exposed to healthcare facilities. Recently, studies have demonstrated that community-acquired CDI is increasing, and is almost 30% of all CDI cases that have so far been reported [12]. In studies conducted by Huang et al., Clabots et al., and Johnson et al., the incidences of CDI rose after a month of hospitalization from 20% to 45.4%, 2.1% to 50%, and 1% to 50%, respectively [13-15]. This could be explained by the fact that C. difficile spores can remain in the atmosphere for months [16]. Infected or colonized patients have a considerably increased number of spores in their stool, and C. difficile are abundantly cultured from the hospital beds, bed rails, floors, and walls which were used by the patients with CDI [17,18]. When new patients occupy the same place, they come in contact with the contaminated environment and acquire C. difficile along with other microorganisms present there [19].

The use of antibiotics and PPI was also found to be significantly correlated with an increased risk of CDI. Studies have suggested that almost every antibiotic results in an increased chance of developing CDI. This also includes the drugs that are used to treat CDI, namely metronidazole and vancomycin. However, broad-spectrum penicillins and cephalosporins, clindamycin, and fluoroquinolones are known to cause higher chances of CDI development compared to other antibiotics [10]. The plausible explanation for this positive correlation is that CDI is initiated with close contact of normal gut flora with antibiotics. This leads to disturbed normal intestinal microflora that provides favorable conditions for C. difficile to multiply and initiate the infection [20]. It is believed that decreasing gastric acid secretion could affect the development of CDI; however, studies could not obtain a satisfactory result to approve this hypothesis. This is supported by the findings that gastric acid could not kill the spores of C. difficile [10]. However, this is controversial because some studies and meta-analyses have proved a significant correlation [21,22], while others could not show a significant association between PPI and CDI risk [23,24]. As mentioned earlier, gastric acid suppressants like PPI and H2RA are associated with an increased risk of CDI. However, in our study, H2RA was not significantly linked with an increased risk of CDI. This could possibly be due to the presence of other risk factors in a considerable number of patients like hospitalization, use of antibiotics, etc.

A higher incidence of CDI was associated with risk factors like increased BMI, diabetes, CKD, and malignancy. In our study, hypertension and hypercholesterolemia did not suggest any correlation with the incidence of CDI. Similar risk factors like CKD, immunosuppression due to malignant neoplasms, transplantations, and inflammatory bowel disease have been reported in previous studies [10,25].

Our study has the following limitations. First it was a single-center study, hence the sample size was limited and less diverse. History of use of antibiotics, proton pump inhibitors and H2RA were given by participants, which may have caused recall bias.

Given the above-mentioned findings, our study suggests that the use of acid-suppressive agents should be carefully considered, and the over-the-counter availability of these agents should be discouraged. This would help the doctors to keep a check in order to avoid overdose. Moreover, hygiene practices such as handwashing practices, sterilizing the used things at the hospital, using clean medical devices, etc. should be adopted to avoid the infection risk. Maximum management of antibiotic intake should also be taken into consideration.

## Conclusions

Our study indicates Clostridium difficile infection (CDI) is associated with older age, recent hospitalization, use of antibiotics, proton pump inhibitors and comorbidities such as malignancy and chronic kidney disease. In recent years, a significant increase in the trends of CDI has been observed. It is important to introduce antibiotic stewardship for rational use of antibiotics, with the help of pharmacies, hospitals and government.
